# The Battle with Cancer when you have a Personality Disorder

**DOI:** 10.1192/j.eurpsy.2025.1925

**Published:** 2025-08-26

**Authors:** A. P. C. Pedro Costa, M. Mousinho, A. Matos Pires, G. Simões, A. I. Gomes, A. R. Coelho

**Affiliations:** 1 Departamento de Saúde Mental, Unidade de Saúde do Baixo Alentejo, Beja; 2 Departamento de Psiquiatria e Saúde Mental, Unidade Local de Saúde da Região de Aveiro, Aveiro; 3 Serviço de Oncologia Médica, nstituto Português de Oncologia de Coimbra Francisco Gentil, Coimbra, Portugal

## Abstract

**Introduction:**

Personality disorders are often comorbid with anxiety and depression disorders, complicating the diagnostic process. Cancer patients with personality disorders who face the anxiety and discomfort associated with the diagnosis, symptoms, and medical treatment may encounter challenges. They might distort reality as a means of emotional self-preservation or display aggression.

**Objectives:**

This review seeks to delve into the challenges in cancer patients exhibiting dysfunctional personality traits or personality disorders.

**Methods:**

A non-systematized literature review was carried out on PubMed and Google Scholar. The following terms were searched: (“personality disorders” OR “personality traits”) AND (“cancer” OR “cancer patients”).

**Results:**

Personality traits persistently influence behavior patterns, choices, environmental interactions, and stress responses. Personality can impact cancer development and progression through multiple avenues: by perpetuating unhealthy lifestyle behaviors rooted in personality traits; through negative affect such as depressive or anxious symptoms, as well as ineffective coping mechanisms; and by serving as an etiological factor for somatic diseases or mental disorders that predispose individuals to cancer. While some epidemiological studies have reported a positive association between personality and cancer development or progression, the majority find no significant correlation, leading researchers to conclude that there is no substantial link between personality and an increased risk of cancer.

**Conclusions:**

Meeting the diverse challenges associated with cancer requires adaptability, flexibility, and resourcefulness. Research suggests that specific personality traits, like neuroticism and negative affectivity, are linked to lower quality of life among cancer patients, while extraversion and optimism are correlated with more favorable outcomes.

**Disclosure of Interest:**

None Declared

